# Lemon Juice in Malaria

**Published:** 1884-11

**Authors:** 


### Lemon Juice in Malaria.

We have already noticed the recommendation of Dr. Tommasi Crudelli, of Rome, in reference to the use of lemon juice in malaria. Before the last International Medical Congress, this Professor gave the following directions for preparing the remedy. A lemon is cut up, peel and all, into thin slices, which are then put into three glassfuls of water, and the whole boiled down to one glassful. It is then strained through linen, squeezing the remains of the boiled lemon, and set aside for some hours to cool. The whole amount of the liquid is then taken fasting. Dr. Mascagni, of Italy, has succeeded with this remedy in curing an obstinate case of malaria in his own person, that had resisted quinine. It is well known that in Italy, Greece, and North Africa; they often use lemon juice or a decoction of lemon seeds, as a remedy in malarial fevers of moderate intensity; and in Guadaloupe they use for the same purpose a decoction of the bark of the roots of the lemon tree. All these popular practices tend to show that the lemon tree produces a febrifuge substance, which resides in all parts of the plant, but which would seem to be most abundant in the fruit. In fact, among the popular remedies employed against malarial infection, this is the most efficacious, for it can be employed with good effects in acute fevers. But it is especially advantageous in combatting the chronic infection, which is rebellious to the action of quinine, and in removing or moderating its deplorable effects.

